# MOZ-Mediated Repression of *p16^INK^^4^^a^* Is Critical for the Self-Renewal of Neural and Hematopoietic Stem Cells

**DOI:** 10.1002/stem.1606

**Published:** 2014-05-23

**Authors:** Flor M Perez-Campo, Guilherme Costa, Michael Lie-a-Ling, Stefano Stifani, Valerie Kouskoff, Georges Lacaud

**Affiliations:** a Cancer Research UK Stem Cell Biology Group, Cancer Research UK Manchester Institute, The University of ManchesterManchester, United Kingdom; b Cancer Research UK Stem Cell Haematopoiesis Group, Cancer Research UK Manchester Institute, The University of ManchesterManchester, United Kingdom; c Montreal Neurological InstituteMontreal, Quebec, Canada

**Keywords:** Hematopoietic stem cell, Neural stem cells, Epigenetics, Histone acetylation, MOZ, Senescence

## Abstract

Although inhibition of *p16^INK4a^* expression is critical to preserve the proliferative capacity of stem cells, the molecular mechanisms responsible for silencing *p16^INK4a^* expression remain poorly characterized. Here, we show that the histone acetyltransferase (HAT) monocytic leukemia zinc finger protein (MOZ) controls the proliferation of both hematopoietic and neural stem cells by modulating the transcriptional repression of *p16^INK4a^*. In the absence of the HAT activity of MOZ, expression of *p16^INK4a^* is upregulated in progenitor and stem cells, inducing an early entrance into replicative senescence. Genetic deletion of *p16^INK4a^* reverses the proliferative defect in both *Moz^HAT^^−^^/^^−^* hematopoietic and neural progenitors. Our results suggest a critical requirement for MOZ HAT activity to silence *p16^INK4a^* expression and to protect stem cells from early entrance into replicative senescence.

## Introduction

Self-renewal of stem cells is vital for maintaining tissues homeostasis throughout the life span of an organism. Because of their high-mitotic activity, stem cells have to put in place inherent cellular defense mechanisms, such as senescence and apoptosis, to avoid the expansion of potentially malignant cells resulting from the accumulation of oncogenic mutations. Cells undergoing senescence display dramatic changes in chromatin structure, which contribute to the irreversible nature of the senescent state. These changes are regulated by the activities of chromatin modifying enzymes; however, the nature of these specific enzymes and their role in the control of senescence remains mostly unknown.

The histone acetyltranferase monocytic leukemia zinc finger protein (MOZ; MYST3 or KAT6A) is a key regulator of hematopoiesis recurrently found translocated in acute myeloid leukemia 1–5. Both MOZ null mouse embryos and mice carrying a G657E mutation, which renders the protein catalytically inactive (*Moz^HAT^^−^^/^^−^* hereafter), have severe defects in the generation and maintenance of hematopoietic stem cells (HSCs) 6–8. In the absence of MOZ histone acetyltransferase (HAT) activity, the proliferative capacity of hematopoietic progenitors is dramatically impaired, with many cells withdrawing from the cell cycle during the G1 phase 8.

In this study, we establish that the proliferative defect observed in the absence of the HAT activity of MOZ is not limited to the hematopoietic compartment, but also extends to neural stem cells and progenitors (NSC/Ps). We show that this proliferative defect is caused by the upregulation of *p16^INK4a^* expression leading to a premature entry into replicative senescence and that the senescent phenotype can be rescued by genetic deletion of *p16^INK4a^*. We further demonstrate that MOZ binds directly to the promoter of *p16^INK4a^* indicating that this tumor suppressor is a direct target of MOZ. Our findings suggest that these two stem cell types, HSCs and NSCs, use the same novel mechanism involving MOZ-driven acetylation to maintain their capacity to proliferate and avoid senescence. Altogether, these results provide new insights into the control of stem and progenitor cell proliferation and identify an unexpected role of MOZ-mediated acetylation in the regulation of *p16^INK4a^* expression. This finding also suggests that a potential reinforcement of the repressive activity of MOZ on *p16^INK4a^* expression could be an important mechanism supporting the development of acute myeloid leukemia following MOZ translocations.

## Materials and Methods

### Cell Culture and Growth Curves

Differentiation of embryonic stem cells (ESCs) into embryoid bodies (EBs) was carried out as described previously 8,9. Serum-free conditions that sustain the proliferation of hematopoietic precursors in liquid culture were described previously 10. For neurospheres culture, we used the “NeuroCult Proliferation Kit” (Stem Cell Technologies, www.stemcell.com). To test the self-renewal capacity of neurospheres, cells were isolated from primary spheres using a NeuroCult Chemical Dissociation Kit (Stem Cell Technologies, www.stemcell.com). Self-renewal was quantified as number of secondary neurospheres generated per primary neurosphere. For proliferation studies, 10 µM 5-bromo-2-deoxyuridine (BrdU) was added to the cultures for 12 hours at 37°C.

### Expression Analysis

Total RNA was extracted with an RNAeasy kit, treated with RNAse-free DNase (QIAGEN, www.qiagen.com), and reverse-transcribed into cDNA with random hexamers by use of an Omniscript RT kit (QIAGEN, www.qiagen.com). Real-time polymerase chain reaction (PCR) was performed on an ABI 7900 system (Applied Biosystems, www.lifetechnologies.com) using the Exiqon universal probe library and primer designer (Roche, www.roche.com). All expression data were calculated relative to β-actin as 2*^−^*^Δct^. Data are presented as ΔCt values from triplicates normalized to β-actin. Primer sequences are available upon request.

### Flow Cytometry

EBs were trypsinized (TryplE; Life technologies, www.lifetechnologies.com) for 3 minutes. Bone marrow of transplanted NOD Scid Gamma NSG mice was isolated by flushing the femurs with phosphate-buffered saline containing 2% fetal bovine serum. Single-cell suspensions were analyzed on a FACScan or a FACScalibur flow cytometer (Becton Dickinson, www.bd.com) or sorted on a FACS Vantage cell sorter (Becton Dickinson). The antibodies used were as follows: Mac1 (biotinylated), Sca-1 labelled with fluorescein isothiocyanate (FITC), and c-Kit (APC) were used for the HSC analysis; For the isolation of Lin*^−^*cKit*^−^* or CD45.2^+^Lin*^−^*cKit^+^ population from bone marrow, we used CD45.2 (Biotin) and cKit labelled with allophycocyanin (APC) antibody together with a mix of antibodies recognizing lineage specific antigens Gr1, Mac1, B220, CD3, and Ter119 (PE). Staining with CD34 (Biotin) and cKit (APC) was performed to isolate hematopoietic progenitors from day 6 EBs. For the isolation of HSCs, we also included a combination of antibodies recognizing members of the SLAM markers CD150 (PE), CD48 (APC), and CD224.2 (FITC). All the antibodies were from BD Pharmingen or ebiosciences. For cell cycle analysis, BrdU incorporation (BrdU Flow kit, BD Pharmingen, www.bdbiosciences.com) was performed according to the manufacturer's instructions.

### Senescence Analysis

Senescence associated β-galactosidase (SA β-gal) assay was performed using a senescence β-galactosidase staining kit (Cell Signaling, www.cellsingal.com).

### Fetal Liver Transplantation and 5FU Treatment

NSG recipients (CD45.1) of 8- to 12-week-old were lethally irradiated with 250cGy in two doses of 125 cGy 3 hours apart and injected with donor (CD45.2) fetal liver cells. To determine the repopulating level of donor cells, peripheral blood was collected and stained with anti-CD45.1 and anti-CD45.2. For analysis of HSC proliferation in vivo, wild type (Wt) and *MOZ^HAT^^−^^/^^−^* mice were intravenously administered 5-fluorouracil (5FU; Mayne Pharma PLC, Warwick, UK) at a single dose of 150 mg/kg body weight. Six days after 5-FU treatment, bone marrow cells were isolated and analyzed for *p16^INK4a^* expression by immunostaining. Sorted 6-day 5FU cells were grown in liquid culture in round-bottom microtiter plates (10 cells per well). After 10 days of incubation, cell number per well was scored using an inverted light microscope.

### Competitive Repopulation Assays

Experimental conditions for this assay were published previously 11. Repopulating units (RUs) from each donor were calculated according to the method described by Harrison and Astle 12, where numbers of RUs are calculated from the percentage donor cells. In brief, the calculations are based in the formula RU = %(C)/(100 − %), where the number of fresh competitor marrow cells used per 10^5^ equals C and percentage corresponds to the obtained percentage of donor cells.

### Transgenic Mice and Embryo Generation

All animal work was performed under regulations governed by the Home Office Legislation under the Animal Scientific Procedures Act of 1986. *Ink4a^+/−^* mice were obtained from Dr. O. Samson with the consent of Dr. M. Serrano.

### ChIP Assays

Chromatin immunoprecipitation was performed using the Red ChIP Kit (Diagenode, www.diagenode.com) following the instructions of the manufacturer. Crosslinked cells were sonicated for 15 cycles (30s on/30s off) with the Bioruptor (Diagenode, www.diagenode.com). Antibodies used were RNA Polymerase (H-224 from Santa Cruz Biotechnology, www.scbt.com) and anti-HAT MYST3 antibody (Ab41718 from Abcam, www.abcam.com). Ten million cells were used for each immunoprecipitation with the anti-Moz antibody. Eluted chromatin was quantified by qualitative PCR (qPCR). Data for ChIP were obtained by subtracting IgG control values to the corresponding antibody values. Graphs represent fold increase over control IgG.

### Immunoblotting and Immunocytochemistry

To analyze protein expression levels, cells were solubilized in Radio-Immunoprecipitation Assay (RIPA) lysis buffer containing a cocktail of protease inhibitors (Sigma Aldrich). Electrophoresis was carried out using commercial reagents (Novex; Life Technologies, www.lifetechnologies.com). For immunoblot, proteins were transferred to a nitrocellulose membrane using the iBlot gel transfer apparatus (Life Technologies, www.lifetechnologies.com). Nonspecific binding was blocked by incubation in blocking buffer; Tris-buffered saline (TBST; 0.1% Tween-20) containing 5% skimmed milk. After incubation with the corresponding secondary antibodies, signal was developed using the Enhanced Chemiluminescence Plus kit (ECL-Plus kit; GE-Healthcare Bio-Sciences, www.gelifesciences.com). For p16^INK^^4^^a^ and HP1-γ immunostainings cells were cytocentrifuged, fixed and stained with the corresponding antibody. Antibodies used were HP1-γ (07332 from Millipore, www.millipore.com) and p16*^INK^^4^^a^* (M-156 from Santa Cruz Biotechnologies, www.scbt.com)

### Statistics

Statistical comparisons of data sets were performed with the two-tailed Student's test.

## Results

### HSC/Ps Undergo Early Entrance into Replicative Senescence in the Absence of MOZ HAT Activity

**Figure 1:**
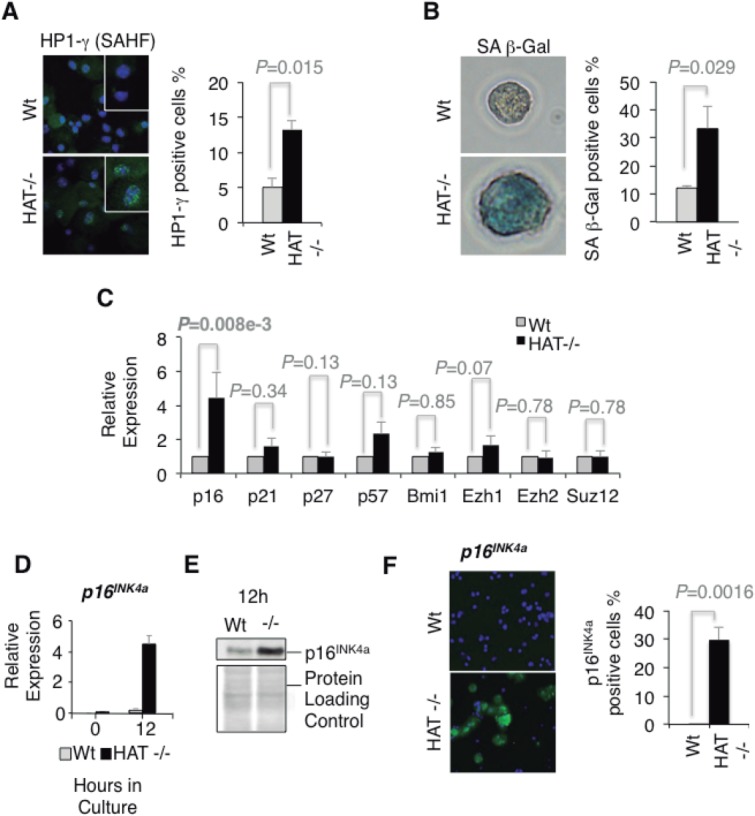
*Moz^HAT^^−^^/^^−^* CD34^+^cKit^+^ hematopoietic progenitors leave the cell cycle to undergo early entrance into replicative senescence. (A): Senescence-associated heterochromatin foci. Wt and *Moz^HAT^^−^^/^^−^* CD34^+^cKit^+^ hematopoietic progenitors isolated from day 6 embryoid bodies (EBs) were stained for Heterochromatin Protein 1-gamma (HP1-γ) (green) and 4',6-diamidino-2-phenylindole (DAPI) (blue) (magnification ×40). Bar graph indicates the percentage of cells stained positive for HP1-γ after 8 days in proliferation media. Bars represent mean ± SEM (B): CD34^+^cKit^+^ hematopoietic progenitors were stained with X-gal to detect the senescence-associated (SA) β-gal activity (pH: 6). Pictures show one representative cell of each group (magnification ×100). Bar graph indicates the percentage of cells stained after 10 days of culture in proliferation media. (C): Qualitative polymerase chain reaction (qPCR) analysis of the expression of diverse cyclin-dependent kinase inhibitors and *p16^INK4a^* regulators in CD34^+^cKit^+^ hematopoietic progenitors. For each gene, values for the *Moz^HAT^^−^^/^^−^* are calculated relative to those of the Wt. Wt and *Moz^HAT^^−^^/^^−^* progenitors cells were directly isolated from day 6 EBs and cultured in proliferation media for 48 hours before the analysis. Graph shows average values of three different experiments. Bars represent mean ± SEM (D): qPCR analysis of *p16^INK4a^* transcripts levels in CD34^+^cKit^+^ cells after 12 hours in proliferation media. (E): Immuno-blot shows p16*^INK4a^* levels of CD34^+^cKit^+^ hematopoietic progenitors 12 hours after plating in proliferation media. (F): Immunostaining of CD34^+^cKit^+^ hematopoietic progenitors 12 hours after plating (magnification ×20). Bar graph indicates the percentage of positive cells for p16^INK4a^. Abbreviations: HAT, histone acetyltransferase; SA β-gal, senescence-associated β-galactosidase; SAHF, senescence-associated heterochromatin foci; Wt, wild type.

**Figure 2:**
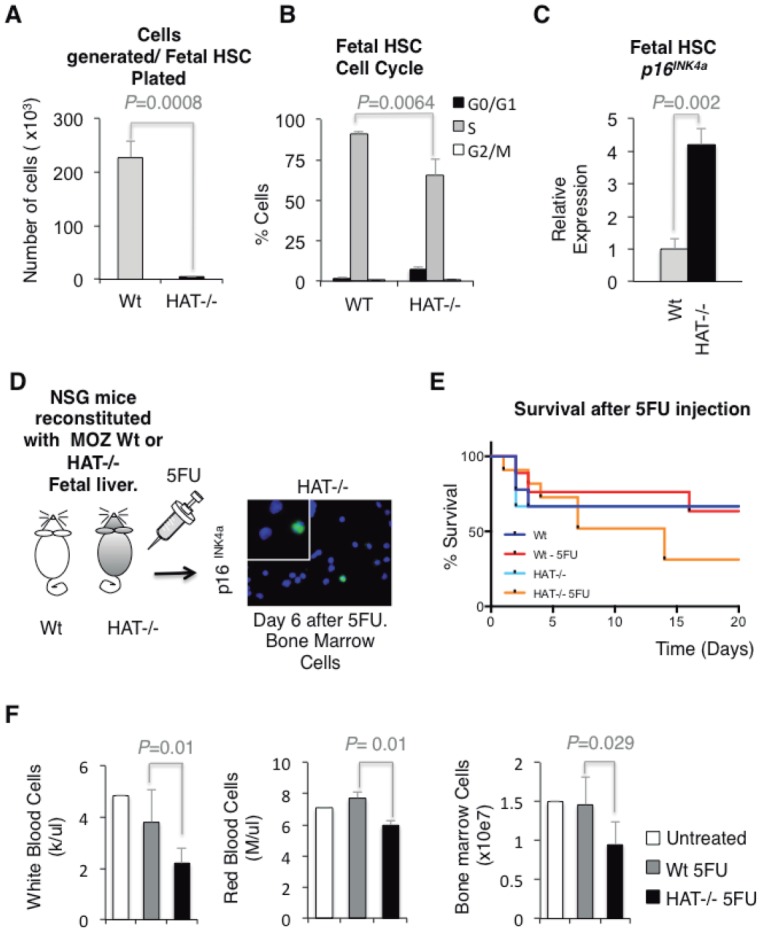
Impaired proliferation and *p16^INK4a^* upregulation in *Moz^HAT^^−^^/^^−^* hematopoietic stem cell (HSC)/Ps in vivo. (A): Individual HSCs (Lin*^−^*Sca^+^cKit^+^CD150^+^CD48*^−^*) isolated from the fetal liver of Wt and *Moz^HAT^^−^^/^^−^* embryos were sorted into 96-well plates containing proliferation media. Cell number was scored after 10 days. (B): Cell cycle status of Lin*^−^*Sca^+^cKit^+^CD150^+^CD48*^−^* fetal liver cells isolated from E14.5 embryos. Pregnant females were injected with 5-bromo-2-deoxyuridine 1 hour before harvesting of embryos and cell cycle was analyzed by flow cytometry. (C): Qualitative polymerase chain reaction analysis of *p16^INK4a^* expression in CD45.2^+^Lin*^−^*Sca^+^cKit^+^CD150^+^CD48*^−^* cells isolated from the bone marrow of reconstituted mice after 24 hours of culture in proliferation media. (D): Reconstituted mice were treated with 5FU. Bone marrow was harvested 6 days later and CD45.2^+^Lin*^−^*cKit^+^ cells were immunostained with a p16^INK4a^ antibody. *MOZ^HAT^^−^^/^^−^* bone marrow cells expressing *p16^INK4a^* are shown in the picture (magnification ×40). No cells positive for the p16^INK4^ staining were detected in the bone marrow of Wt reconstituted mice (data not shown). (E): Kaplan-Meier graph showing the survival of reconstituted NSG mice after 5FU injection. Control mice were not injected. Wt (*n* = 6), Wt 5FU (*n* = 6), histone acetyltransferase HAT^−/−^ (*n* = 8) and HAT^−/−^ 5FU (*n* = 8). (F): Low white and red blood cell counts and reduced bone marrow cellularity are detected in NSG mice reconstituted with *Moz^HAT^^−^^/^^−^* fetal liver cells 7 days after injection with 5FU. Abbreviations: HAT, histone acetyltransferase; HSC, hematopoietic stem cell; MOZ, monocytic leukemia zinc finger protein; NSG, NOD Scid Gamma; Wt, wild type.

We established previously that HSCs and blood precursors carrying the mutated G657E MOZ protein lacking HAT activity have a profound proliferative deficiency, with many cells arresting in the G1 phase of the cell cycle 8. A cell leaving the cell cycle during the G1 phase may encounter different fates: it can differentiate, become quiescent, senescent, or undergo apoptosis. No signs of increased apoptosis or defects in differentiation were observed in *Moz^HAT^^−^^/^^−^* hematopoietic progenitors 8 suggesting quiescence or senescence as the most likely fates. Therefore, we evaluated whether the reported G1 arrest previously observed in *Moz^HAT^^−^^/^^−^* CD34^+^cKit^+^ hematopoietic progenitors is related to the acquisition of a senescent phenotype. Consistent with this hypothesis, a higher frequency of cells positive for the presence of senescence-associated heterochromatin foci (SAHF) 13, marking the accumulation of the nuclear Heterochromatin Protein 1-gamma (HP1γ) protein was detected in *Moz^HAT^^−^^/^^−^* CD34^+^cKit^+^ hematopoietic progenitors generated by in vitro ESC differentiation than in their Wt counterparts (Fig. 1A). A significantly higher percentage of the hematopoietic progenitors also expressed the senescence-associated β-galactosidase (SA β-gal) in the absence of the HAT activity of MOZ (Fig. 1B). The progression through the G1 phase of the cell cycle in stem cells has been shown to be controlled by different cyclin-dependent kinase (CDK) inhibitors, such as p16(*INK4a*) 14–16, p21(CIP1) 16–20, p27 (Kip1) 21,22, and p57(Kip2) 23,24. We analyzed the transcription levels of these CDK inhibitors to evaluate whether changes in their expression could be linked to the observed phenotype in *Moz^HAT^^−^^/^^−^* hematopoietic progenitors. Only transcriptional levels of the tumor suppressor *p16^INK4a^* were significantly altered in *Moz^HAT^^−^^/^^−^* cells (Fig. 1C). To further investigate whether this upregulation of *p16^INK4a^* was reflecting changes in the transcription levels of known regulators of this tumor suppressor, such as Bmi1 15,25, Ezh1 26, Ezh2 27,28, and Suz12 29, we analyzed the expression of these genes by qPCR. We found no significant difference in the expression levels of proteins known to control *p16^INK4a^* transcription (Fig. 1C). We then verified that the transcription levels of *p16^INK4a^* were rapidly upregulated in *Moz^HAT^^−^^/^^−^* hematopoietic progenitors upon culture conditions that promote their proliferation (Fig. 1D). Higher levels of p16*^INK^^4^^a^* protein were also detected in these cells by immunoblotting and immunostaining (Fig. 1E, 1F). Similarly, to the in vitro ESC-derived blood cells, *Moz^HAT^^−^^/^^−^* cells isolated from embryonic fetal liver and highly enriched for HSCs (Lin*^−^*Sca^+^cKit^+^CD150^+^CD48*^−^*) 30 had a limited proliferative capacity (Fig. 2A). This proliferative defect was reflected by a significantly lower percentage of cells in the S-phase of the cell cycle, and the accumulation of cells in the G1 phase as shown by BrdU incorporation analysis (Fig. 2B). qPCR analysis also revealed that *Moz^HAT^^−^^/^^−^* fetal liver HSCs displayed increased expression levels of *p16^INK4a^* upon culture (Fig. 2C). To confirm our findings with adult hematopoietic progenitors and circumvent the limiting perinatal lethality of *Moz^HAT^^−^^/^^−^* mice, we transplanted Wt or *Moz^HAT^^−^^/^^−^* fetal liver cells (CD45.2^+^) into irradiated immunodeficient NSG (CD45.1^+^) mice. Analyses of peripheral blood chimerism in transplanted animals 4 weeks after transplantation indicated that baselines of engraftment by CD45.2^+^ cells were higher than 90% and similar between mice repopulated by either Wt or *Moz^HAT^^−^^/^^−^* fetal liver cells (Supporting Information Fig. S1A). We then isolated adult CD45.2^+^Lin*^−^*cKit^+^ hematopoietic progenitors from the bone marrow of the reconstituted mice for analysis. The *Moz^HAT^^−^^/^^−^* cells displayed again proliferative defects associated with an increase in p16^INK^^4^^a^ protein levels upon ex vivo culture in proliferation media (Supporting Information Fig. S1B–S1F). To confirm these ex vivo findings and assess proliferation in vivo, cohorts of reconstituted mice were treated with 5FU to induce HSC entry into cell cycle 31,32. The p16^INK^^4^^a^ protein was detected by immunostaining in CD45.2^+^Lin*^−^*cKit^+^ bone marrow cells isolated from 5FU treated *Moz^HAT^^−^^/^^−^* mice 6 days after treatment (Fig. 2D), whereas no positive staining was observed in 5FU-treated Wt controls. In addition, 7–10 days after treatment, a high percentage of 5FU-treated mice reconstituted with *Moz^HAT^^−^^/^^−^* had to be euthanized (Fig. 2E) due to excessive weight loss (Supporting Information Fig. S1G) associated with the development of low blood counts (Fig. 2F). These findings are consistent with the profound long-term repopulation potential defect of *Moz^HAT^^−^^/^^−^* HSCs in serial transplantation experiments as documented previously 8. Altogether, these experiments demonstrate that the absence of the HAT activity of MOZ either in ESCs derived, fetal or adult hematopoietic progenitors results in cell autonomous proliferative defects triggered by a premature entry into replicative senescence.

### NSCs Self-Renewal Relies on MOZ HAT Dependent Silencing of *p16^INK4a^*

**Figure 3:**
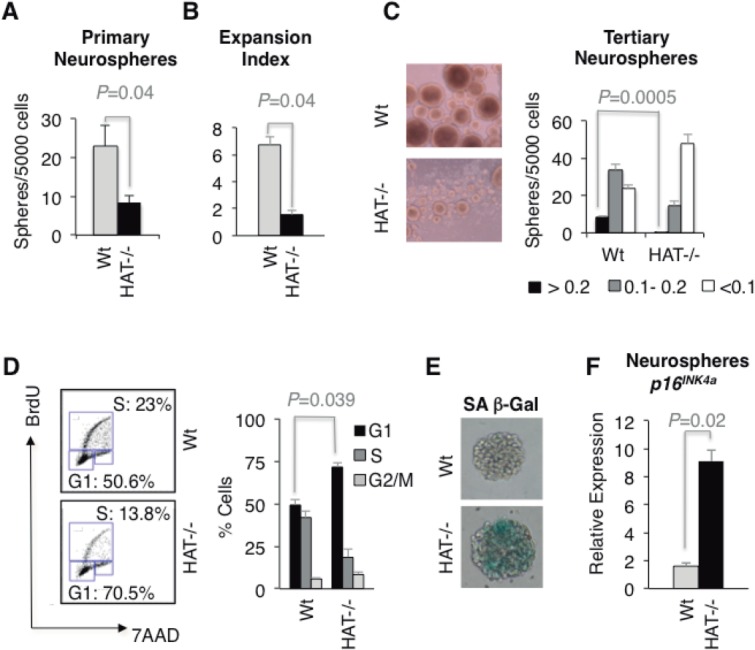
Telencephalon cells isolated from *Moz^HAT^^−^^/^^−^* embryos display marked proliferative defects and signs of replicative senescence. (A): Frequency of primary neurospheres generated by E14.5 telencephalon cells. (B): Expansion index indicating the number of secondary neurospheres generated per primary neurosphere. (C): Morphology of Wt and *Moz^HAT^^−^^/^^−^* day 8 tertiary neurospheres. Neurospheres are classified in different groups based on the diameter of the sphere. (<0.1 mm, 0.1–0.2 mm, and >0.2 mm). Bars represent mean ± SEM (*n* = 4). (D): Cell-cycle status of cells in neurospheres. Cells were isolated from day 6 secondary neurospheres. Percentage of cells in G1, S, and G2/M phases are indicated. Bars represent mean ± SEM (*n* = 3). (E): Senescence-associated β-galactosidase activity (pH: 6) in day 7 Wt and *Moz^HAT^^−^^/^^−^* primary neurospheres. Magnification (×40). (F): Analysis of *p16^INK4a^* transcript levels in day 7 Wt, and *Moz^HAT^^−^^/^^−^* neurospheres (*n* = 4 for each genotype). Abbreviations: 7AAD, 7-Aminoactinomycin D; BrdU, 5-bromo-2-deoxyuridine; HAT, histone acetyltransferase; SA β-gal, senescence-associated β-galactosidase; Wt, wild type.

**Figure 4:**
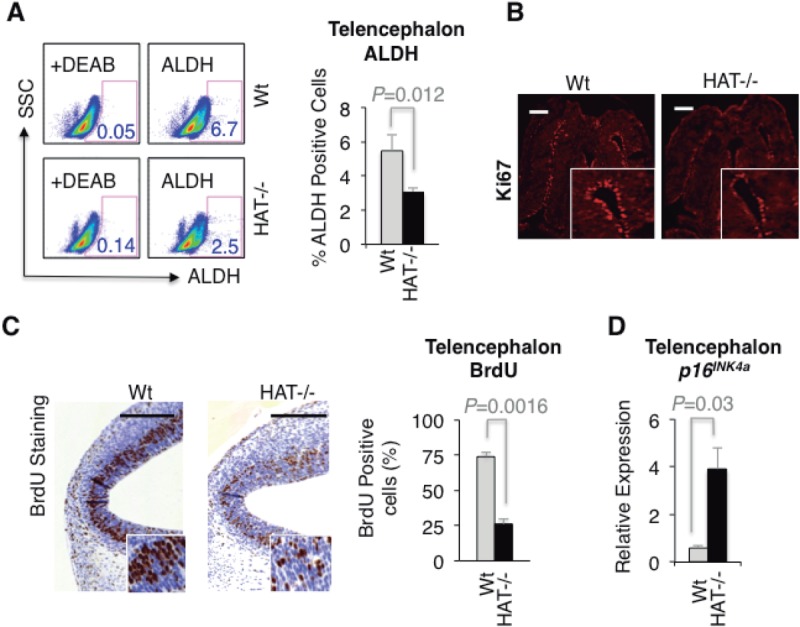
Impaired proliferation and *p16^INK4a^* upregulation in *Moz^HAT^^−^^/^^−^* neural stem cells and progenitors in vivo. (A): Flow cytometry plots of ALDH activity in Wt and *Moz^HAT^^−^^/^^−^* telencephalon cells. Cells isolated from E14.5 telencephalons were incubated with the ALDH substrate aldefluor in the presence or absence of the ALDH inhibitor diethylaminobenzaldehyde. Bar graph reflects the average percentage of ALDH positive cells. (B): Coronal sections of Wt and *Moz^HAT^^−^^/^^−^* E14.5 telencephalons stained with anti-Ki67 antibody. Scale bar = 0.2 mm. (C): Sections of Wt and *Moz^HAT^^−^^/^^−^* E14.5 telencephalons stained with anti-BrdU antibody. Bar graph represents the percentage of cells stained with BrdU. (D): Analysis of *p16^INK4a^* transcript levels in telencephalon tissue (*n* = 24) isolated from Wt and *Moz^HAT^^−^^/^^−^* E14.5 embryos. Scale bar = 0.2 mm. Abbreviations: ALDH, aldehyde dehydrogenase; BrdU, 5-bromo-2-deoxyuridine; DEAB, diethylaminobenzaldehyde; HAT, histone acetyltransferase; SSC, Side Scatter; Wt, wild type.

MOZ and its close homologue MORF (MOZ related factor, MYST4 or KAT6B) have been assigned specific roles in either hematopoietic or neural development, respectively 33,34. However, our previous observation that *Moz^HAT^^−^^/^^−^* ESCs, unlike their Wt counterparts, did not extensively contribute to the formation of the brain in chimeric mice 8 suggests that MOZ, through its HAT activity, might also play a role in regulating the proliferation of NCS/Ps. To test this hypothesis, we first cultured cells isolated from the telencephalon of Wt and *Moz^HAT^^−^^/^^−^* E14.5 embryos under clonogenic conditions to compare their potential to generate self-renewing neurospheres, a measurement of the number of cells with neural stem-like properties 35,36. *Moz^HAT^^−^^/^^−^* embryos generated three times less neurospheres than Wt controls (Fig. 3A) suggesting that there are significantly fewer NSCs in the telencephalon of *Moz^HAT^^−^^/^^−^* embryos. In addition, *Moz^HAT^^−^^/^^−^* neurospheres displayed reduced expansion kinetics, producing fewer neurospheres at each passage, with an expansion index fivefold lower than Wt neurospheres (Fig. 3B), suggesting a reduced self-renewal potential. In fact, no neurospheres could be generated from the *MOZ^HAT^^−^*^/^*^−^* cells after the third passage. In addition to this reduced expansion rate, *Moz^HAT^^−^^/^^−^* neurospheres were, on average, smaller than their Wt counterparts (Fig. 3C), which could be indicative of cell-cycle arrest. BrdU incorporation analysis of *Moz^HAT^^−^^/^^−^* secondary neurospheres revealed a reduced percentage of cells in the S-phase of the cell cycle and accumulation of cells at the G1 phase, similar to the phenotype observed for *Moz^HAT^^−^^/^^−^* hematopoietic progenitors (Fig. 3D). Consistent with the acquisition of a senescent phenotype, cells from secondary *Moz^HAT^^−^^/^^−^* neurospheres also displayed SA β-gal activity and higher *p16^INK4a^* expression levels than Wt controls (Fig. 3E, 3F). These in vitro findings were further substantiated in vivo by the observation that a lower percentage of cells expressing aldehyde dehydrogenase (ALDH), a marker of cells with stem-like properties 37,38, was detected in brains of E14.5 *Moz^HAT^^−^^/^^−^* embryos compared with Wt embryos (Fig. 4A). Furthermore, a reduction in the number of cells expressing the proliferation marker Ki67 (Fig. 4B) and cells incorporating BrdU (Fig. 4C) was also observed in the brains of E14.5 *Moz^HAT^^−^^/^^−^* embryos. Finally, qPCR analysis of E14.5 telencephalons revealed an increased *p16^INK4a^* expression in the telencephalon of *Moz^HAT^^−^^/^^−^* embryos (Fig. 4D). Altogether, these data demonstrate that, similarly to the hematopoietic system, *Moz^HAT^^−^^/^^−^* NSC/Ps display proliferative defects ex vivo and in vivo, upregulate the expression of *p16^INK4a^* and readily enter into replicative senescence.

### Genetic Deletion of *p16^INK4a^* Largely Restores the Proliferative Capacity of HSC/Ps and NSC/Ps

**Figure 5:**
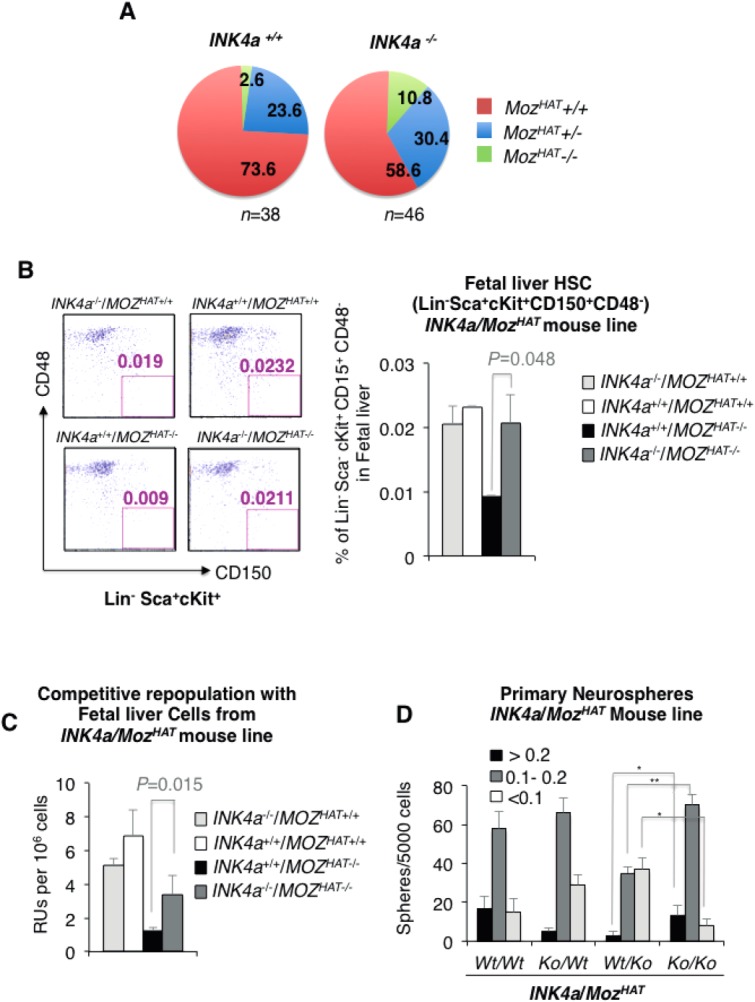
Impaired proliferation of *Moz^HAT^^−^^/^^−^* hematopoietic stem cell (HSC), neural stem cell, and progenitors is rescued by genetic deletion of *p16^INK4a^*. (A): Genotype segregation of live mice produced by intercrossing of heterozygotes mice for the *p16^INK4a^*/*ARF* and *Moz^HAT^* mutations (*Ink4a^+/−^*, *Moz^HAT^^+/^^−^*). Numbers indicate the frequencies of the mice for each genotype. (B): Analysis of E14.5 fetal liver HSCs frequencies in *INK4a*^−/−^*Moz^HAT^^+/^^+^* (Ko/Wt), *INK4a*^+/+^*Moz^HAT^*^+/+^ (Wt/Wt), *INK4a*^+/+^*Moz^HAT^^−^^/^^−^* (Wt/Ko), and *INK4a*^−/−^*Moz^HAT^^−^^/^^−^* (Ko/Ko) littermates. Fluorescence-activated cell sorting plots show the percentage of Lin*^−^*Sca^+^cKit^+^ CD150^+^CD48*^−^* cells of representative individuals in each group. Bar graph represents the average percentage of Lin*^−^*Sca^+^cKit^+^ CD150^+^CD48*^−^* cells in each phenotype. (C): Competitive repopulation assay of irradiated mice. Data are expressed as Repopulating units per 10e6 cells. (D): Bar graph shows the size of the neurospheres formed by telencephalon cells isolated from *INK4a*^+/+^*Moz^HAT^^+/^^+^* (Wt/Wt), *INK4a*^−/−^*Moz^HAT^^+/^^+^*(Ko/Wt), *INK4a*^+/+^*Moz^HAT^^−^^/^^−^* (Wt/Ko), and *INK4a*^−/−^*Moz^HAT^^−^^/^^−^* (Ko/Ko) littermates. *p < .05; **p < .01. Abbreviations: HSC, hematopoietic stem cell; Ko, knockout; MOZ, monocytic leukemia zinc finger protein; RU, repopulating unit; Wt, wild type.

As *p16^INK4a^* upregulation could be exacerbated in culture, we decided to directly evaluate to which extent the proliferative defects observed in *Moz^HAT^^−^^/^^−^* mice are associated to an entry into replicative senescence induced by *p16^INK4a^* upregulation in vivo. To this end, we crossed heterozygote mice for the HAT mutation with *p16^INK4a^*/*p19^ARF^* knockout mice (hereafter *INK4a*^−/−^) 39 to generate double-knockout mice as well as heterozygotes and Wt control littermates. In the absence of *INK4a*, the embryonic and perinatal lethality of *Moz^HAT^^−^^/^^−^* mice was clearly diminished resulting in increased frequency of *Moz^HAT^^−^^/^^−^* mice at weaning (Fig. 5A). In addition, in the absence of *p16^INK4a^*, *Moz^HAT^^−^^/^^−^* mice displayed an overall improved health status and a recovery of the runt phenotype reported previously for these mice 8. We next investigated whether the decreased frequency in HSC population observed in the fetal liver of *Moz^HAT^^−^^/^^−^* embryos 8 was restored to normal level by deletion of *p16^INK^^4^*. Indeed *INK4a*^+/+^/*Moz^HAT^^−^*^/^*^−^* (Wt/Ko) embryos displayed a reduced percentage of HSCs (Lin*^−^*Sca^+^cKit^+^CD150^+^CD48*^−^*), whereas the frequency of these cells was significantly higher in *Moz^HAT^^−^^/^^−^* mice lacking *p16^INK4a^* (Ko/Ko) reaching similar values to those detected in *INK4a*^+/+^/*Moz^HAT+/+^* (Wt/Wt) embryos (Fig. 5B). To further investigate the contribution of p16^INK^^4^^a^ to the proliferative defects of *Moz^HAT^^−^^/^^−^* HSCs, we evaluated the ability of *Moz^HAT^^−^^/^^−^* fetal liver cells on different *INK4a* backgrounds to repopulate the bone marrow of lethally irradiated recipients. *INK4a*^+/+^/*Moz^HAT+/+^* (Wt/Wt), *INK4a*^−/−^/*Moz^HAT+/+^* (Ko/Wt), *INK4a*^+/+^/*Moz^HAT^^−^*^/^*^−^* (Wt/Ko) and *INK4a*^−/−^/*Moz^HAT^^−^*^/^*^−^* (Ko/Ko) E14.5 CD45.2^+^ fetal liver cells were transplanted into lethally irradiated congenic CD45.1^+^ mice together with competitor CD45.1^+^/CD45.2^+^ cells. *INK4a*^−/−^/*Moz^HAT^^−^*^/^*^−^* (Ko/Ko) fetal liver cells showed a significantly higher capacity to repopulate the bone marrow of recipient mice than *INK4a*^+/+^/*Moz^HAT^^−^*^/^*^−^* (Wt/Ko) cells (Fig. 5C) indicating that the defective self-renewal capacity of *Moz^HAT^^−^^/^^−^* HSCs could be rescued, at least partially, by the deletion of *p16^INK4a^* and strongly suggesting that *p16^INK4a^* is a critical MOZ target for the maintenance of HSC self-renewal. Similar to what we observed for the hematopoietic system, the genetic deletion of *p16^INK4a^* resulted in higher numbers of primary neurospheres generated by telencephalon cells of E14.5 *Moz^HAT^^−^^/^^−^* embryos (Fig. 5D). Furthermore, serial passage of these primary neurospheres produced numbers of tertiary neurospheres similar of that of the Wt/Wt (Supporting Information Fig. S2A), indicating a substantial recovery of the expansion index of *Moz^HAT^^−^^/^^−^* neurospheres in the absence of *INK4a* (Supporting Information Fig. S2B). Altogether, these results suggest that the defects observed in these *Moz^HAT^^−^^/^^−^* neural cells are also mediated by the upregulation of *p16^INK4a^*. These results indicate that *p16^INK4a^* upregulation in *Moz^HAT^^−^^/^^−^* cells inhibit NSC self-renewal driving cells into replicative senescence in a similar fashion to HSC/Ps lacking MOZ HAT activity.

### MOZ Binds to the Promoter of the *p16^INK4a^* Tumor Suppressor

In the absence of significant changes in the expression levels of known *p16^INK4a^* regulators, we decided to evaluate next by chromatin immunoprecipitation whether MOZ could directly bind to the *p16^INK4a^* promoter. The use of hematopoietic progenitors for these experiments would involve the isolation of very large number of cells difficult to obtain. To overcome this limitation, we decided to check whether the senescent phenotype was conserved in *Moz^HAT^^−^^/^^−^* mouse embryonic fibroblasts (MEFs), which would provide a source of large numbers of cells needed for this study. We observed in clonogenic assays that the number of large proliferative colonies formed by individual *MOZ^HAT^^−^^/^^−^* MEFs was almost three times less than those formed by Wt MEFs (Supporting Information Fig. S3A). Additionally, growth curves and cell cycle analyses using BrdU revealed a significant decline in the proliferation rate of the *Moz^HAT^^−^^/^^−^* population over time (Supporting Information Fig. S3B) as well as a defect in the progression into the S-phase of the cell cycle (Supporting Information Fig. S3C). *Moz^HAT^^−^^/^^−^* MEFs at passage five showed a high proportion of flattened cells containing SA-β-gal (Supporting Information Fig. S3D) and qPCR analysis of *p16^INK4a^* expression revealed that transcript levels were upregulated on average threefold in *Moz^HAT^^−^^/^^−^* MEFs compared with Wt (Supporting Information Fig. S3E). Accordingly, protein levels of p16*^INK^^4^^a^* were clearly higher in *Moz^HAT^^−^^/^^−^* MEFs than in the Wt and heterozygous *Moz^HAT^^+/^^−^* MEFs (Supporting Information Fig. S3F). Together these results clearly indicate that the senescent phenotype mediated by the upregulation of *p16^INK4a^* was also observed in *Moz^HAT^^−^^/^^−^* MEFs.

**Figure 6:**
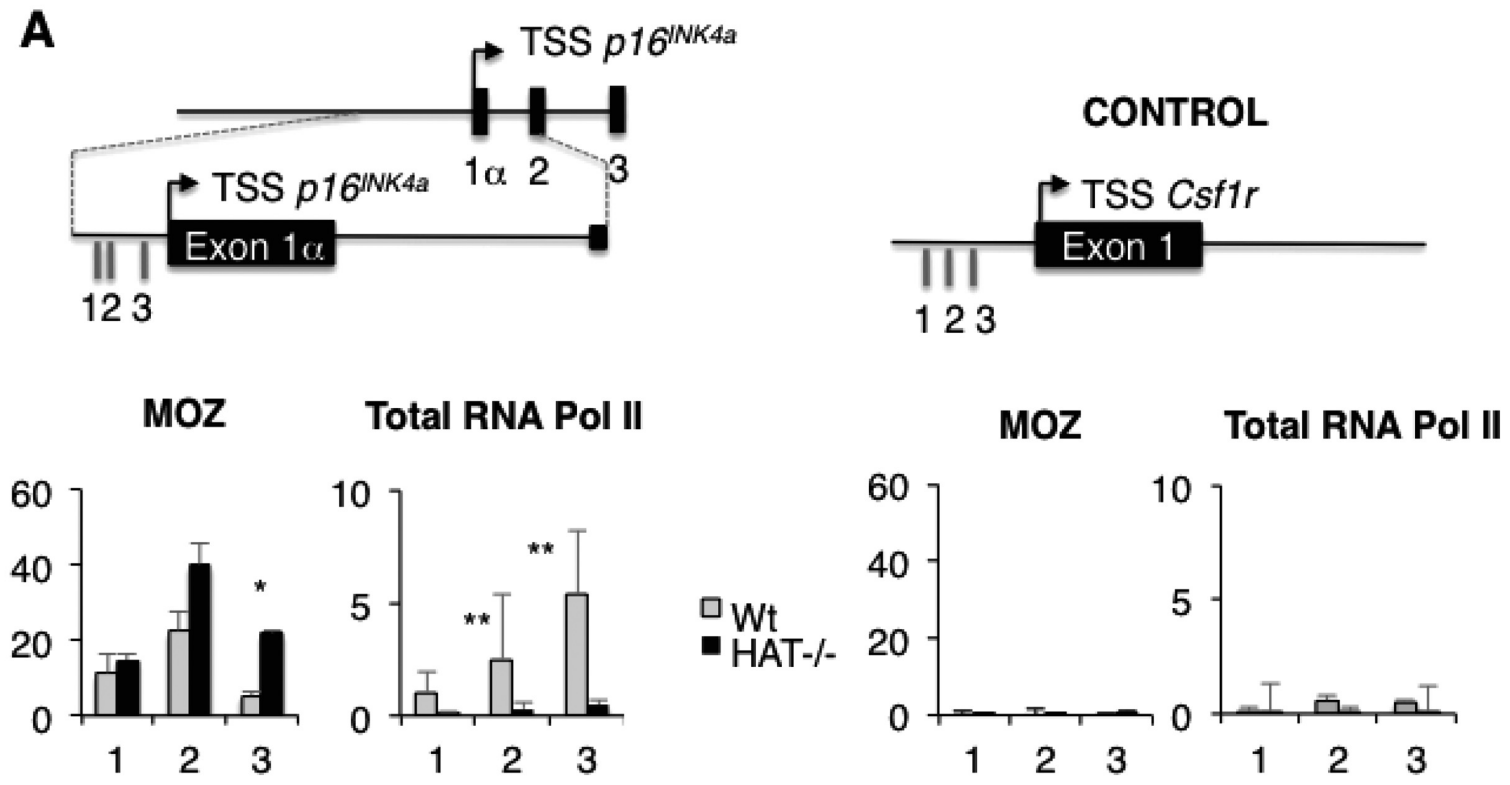
Monocytic leukemia zinc finger protein (MOZ) binding to the *p16^INK4a^* promoter at Wt and *MOZ^HAT^^−^^/^^−^* Mouse embryonic fibroblasts. (A): Upper panel depicts *p16^INK4a^* promoter, exons, and introns. Numbers indicate amplified regions. Lower panel shows the ChIP analysis of MOZ binding at the *p16^INK4a^* promoter in Wt and *Moz^HAT^^−^^/^^−^* MEFs. Binding of RNA Polymerase II was used as a positive control. Samples were prepared from passage 3 MEFs. The promoter of the CSF1R gene, not expressed in MEFs, was used as a negative control. *p < .05; **p < .01. Abbreviations: MOZ, monocytic leukemia zinc finger protein; TSS, Transcription Start Site.

To determine whether the transcriptional upregulation of *p16^INK4a^* in the *Moz^HAT^^−^^/^^−^* cells could be mediated by direct binding of MOZ to this locus, we performed ChIP analyses using MEFs isolated from E13.5 embryos. We detected MOZ binding to the *p16^INK4a^* promoter in Wt MEFs. This binding was conserved in *Moz^HAT^^−^^/^^−^* MEFs indicating that the HAT mutation has no impact on the ability of MOZ to bind to this locus (Fig. 6A). A slightly higher binding frequency of MOZ to the *p16^INK4a^* promoter in the absence of HAT activity was consistently observed and might reflect a compensatory mechanism. Altogether, these results indicate that MOZ directly binds to the *p16^INK4a^* promoter. This might through acetylation of histones or potentially other interacting proteins, repress the transcription of this locus.

## Discussion

**Figure 7:**
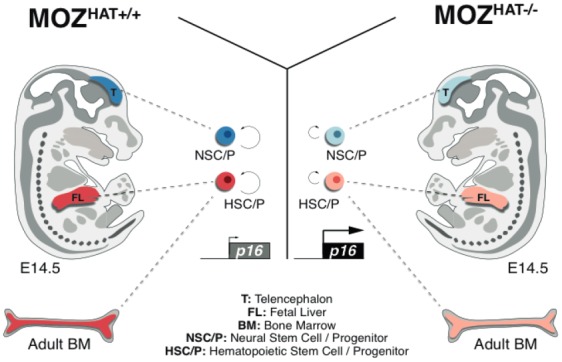
Monocytic leukemia zinc finger protein (MOZ) histone acetyltransferase (HAT) activity regulates the proliferation of different types of stem cells. The histone acetyltrasferase activity of MOZ prevents entry into early replicative senescence by regulating the expression of the tumor supperssor *p16^INK4a^*. In the absence of MOZ HAT activity, the levels of *p16^INK4a^* are significantly increased in both hematopoietic and neural stem and progenitor cell compartments. These cells then leave the cell cycle to become senescent, therefore resulting in severely impaired hematopoietic stem cells and neural stem cells self-renewal. Abbreviations: BM, bone marrow; HAT, histone acetyltransferase; HSC, hematopoietic stem cell; MOZ, monocytic leukemia zinc finger protein; NSC, neural stem cell.

In this study, we establish that in the absence of the HAT activity of MOZ HSC/Ps readily exit the cell cycle to undergo premature entry into replicative senescence. These findings provide a likely explanation for the reported impairment of HSC self-renewal observed in mice expressing the catalytically inactive version of MOZ 8. In contrast to previous studies restricting the critical functions of MOZ and its close homologue MORF to hematopoietic and NSCs, respectively 34, we demonstrate here that similar proliferative defects are found in NSC/Ps lacking MOZ HAT activity (model in Fig. 7). Our data reveal that this common phenotype is at least partially caused by a premature upregulation of *p16^INK4a^* expression. Accordingly, genetic deletion of *p16^INK4a^* rescues to a large extent the proliferative defect. The fact that this phenotype is shared between the hematopoietic and neural compartments suggests that MOZ controls a regulatory mechanism conserved among stem cells from different tissues. This notion is supported by previous results showing that the contribution to specific organs in chimeric mice was consistently lower with *Moz^HAT^^−^^/^^−^* ESCs than with Wt ESCs. The tissues with different contributions also included gut and liver in addition to the brain and hematopoietic organs 8.

The hematopoietic and neuronal phenotype of the *Moz^HAT^^−^^/^^−^* mice bear strong similarities with the proliferative defects and premature senescence observed in neuronal and hematopoietic cells in the Bmi1 knock out animals 25,36,40–43. Bmi1 and other polycomb members are well-established negative epigenetic regulators of *p16^INK4a^* 44 whereas thritorax proteins 45 and SWI/SNF proteins 46 act positively on its expression. We did not detect a significant change in the level of expression of the *p16^INK4a^* regulators analyzed. However, we observed a direct interaction of MOZ to *p16^INK4a^* promoter suggesting that MOZ could introduce changes in histone acetylation pattern which, in turn, could alter the binding of transcriptional regulators of *p16^INK4a^* harboring bromodomains. Further studies will be required to determine whether the HAT activity of MOZ directly impacts on the binding of regulators of *p16^INK4a^* expression or whether MOZ is implicated in a completely novel level of regulation. The *INK4a* locus is one of the genomic regions most commonly mutated, deleted or epigenetically silenced in human cancers 47,48. It has been proposed that the fusion proteins produced upon translocation of the human *MOZ* locus with other HAT-encoding genes, such as CBP or p300, support the development of leukemia by altering the regulation of MOZ transcriptional targets. It would be interesting to examine if the repressive activity on *p16^INK4a^* expression mediated by MOZ acetylation is further exacerbated in these fusion proteins. As such, these MOZ leukemic fusion proteins might inhibit the triggering of senescence and promote the development of leukemia 49. Our findings also raise the intriguing possibility that the regulation of *p16^INK4a^* expression by MOZ could be used as a molecular target to induce senescence in cancer stem cells.

## Conclusion

The histone acetyltranferase MOZ (Monocytic Leukemia Zinc Finger protein, MYST3, or KAT6A) has a crucial role in controlling hematopoietic stem cells (HSCs) proliferation. In this study, we identified a critical requirement for MOZ-HAT activity to silence *p16^Ink4a^* expression, to avoid senescence and sustain self-renewal of hematopoietic stem cells. We established that this effect is not limited to the hematopoietic compartment, but extends to neural stem cells and progenitors (NSC/P) suggesting that these two types of cells, HSCs and NSCs use the same mechanism involving MOZ-driven acetylation in order to maintain their capacity to proliferate. We propose that this mechanism could be also be critical for the self renewal of other types of stem cells.

## Supporting Information

See www.StemCells.com for supporting information available online.
