# Supplementary figures and images for: MOZ-Mediated Repression of p16INK4a Is Critical for the Self-Renewal of Neural and Hematopoietic Stem Cells
Source: Stem Cells. 2014 May 23;32(6):1591–601. doi: 10.1002/stem.1606 (PMC4237135; doi:10.1002/stem.1606)

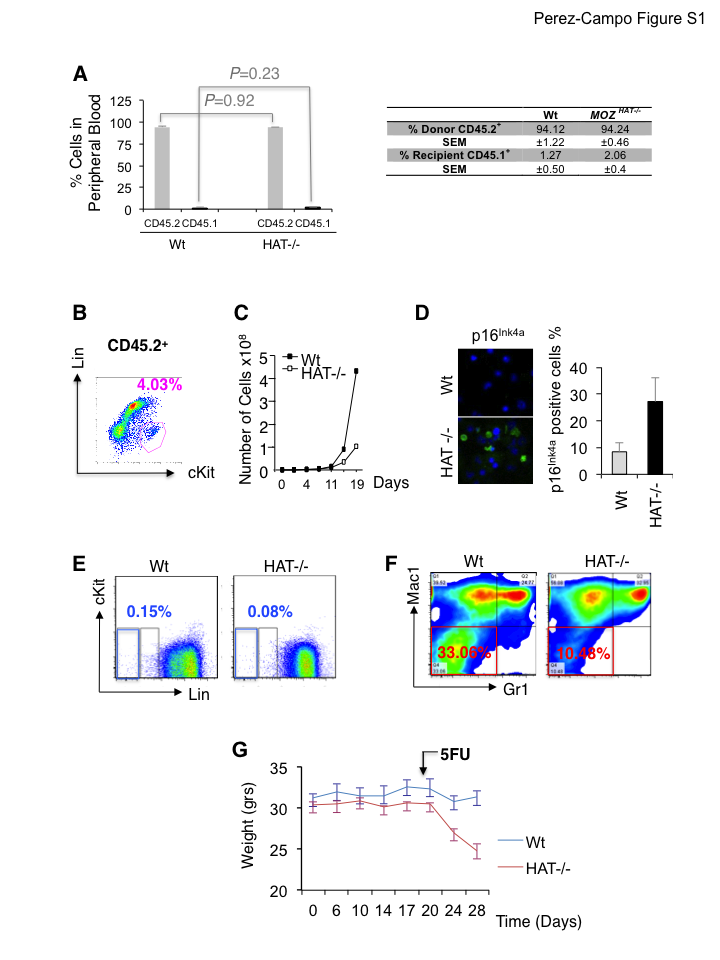

Supplement: Supplementary file 1 [file stem0032-1591-sd1.tiff]

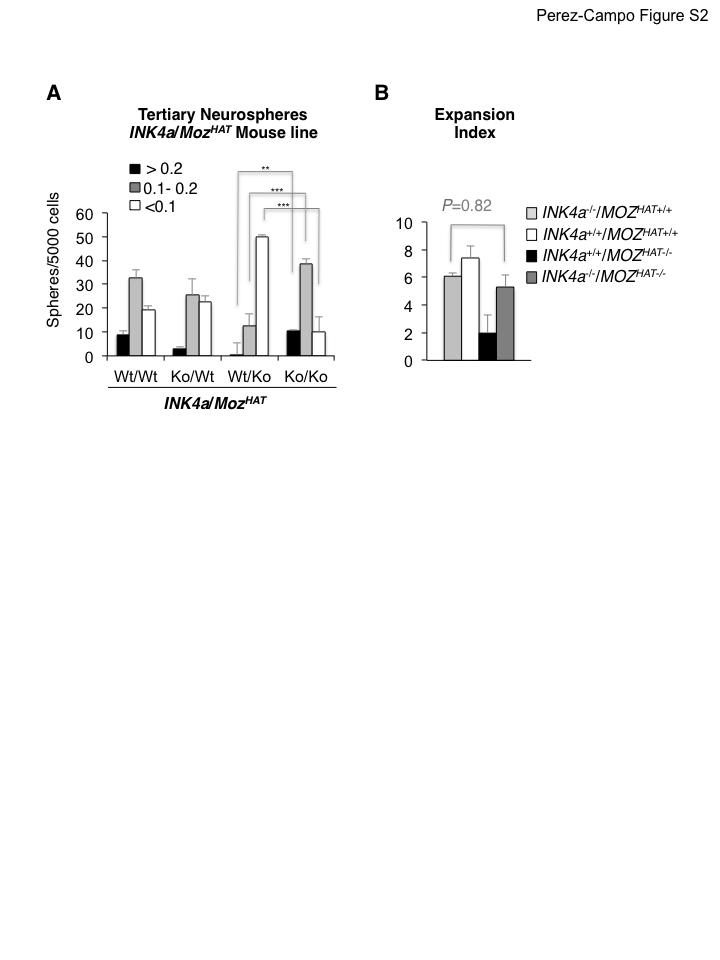

Supplement: Supplementary file 2 [file stem0032-1591-sd2.tiff]

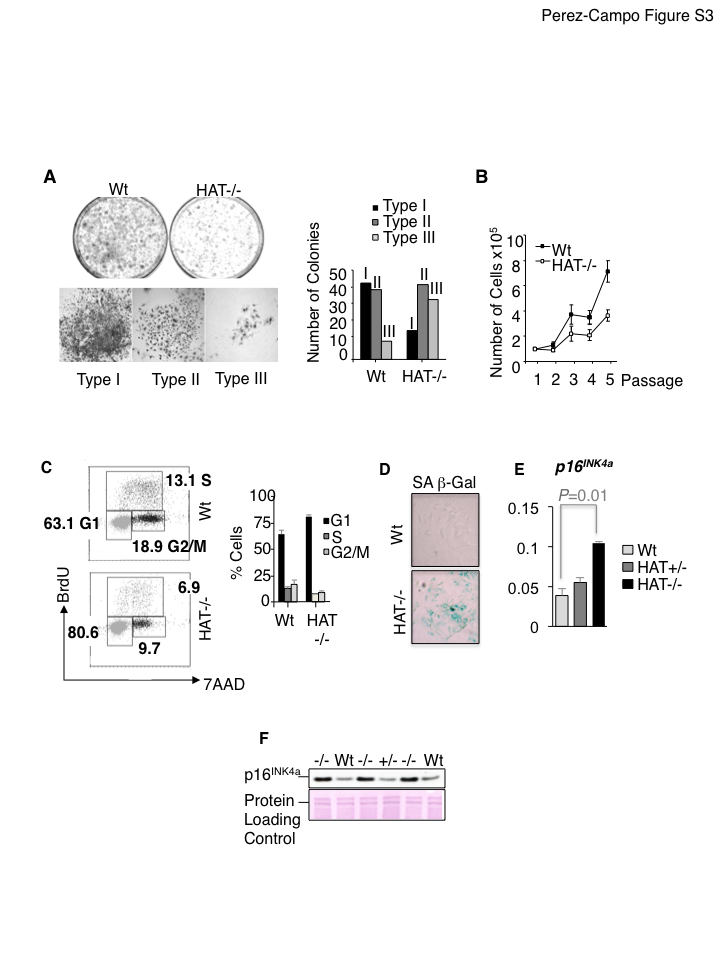

Supplement: Supplementary file 3 [file stem0032-1591-sd3.tiff]
